# *Clostridium difficile* with Moxifloxacin/Clindamycin Resistance in Vegetables in Ohio, USA, and Prevalence Meta-Analysis

**DOI:** 10.1155/2014/158601

**Published:** 2014-12-14

**Authors:** Alex Rodriguez-Palacios, Sanja Ilic, Jeffrey T. LeJeune

**Affiliations:** ^1^Division of Gastroenterology and Liver Disease, Digestive Research Center, Case Western Reserve University School of Medicine, Cleveland, OH 44106, USA; ^2^Department of Human Nutrition, College of Human Nutrition and Human Sciences, The Ohio State University, Columbus, OH 43210, USA; ^3^Food Animal Health Research Program, Ohio Agricultural Research and Development Center, The Ohio State University, Wooster, OH 44691, USA

## Abstract

We (i) determined the prevalence of *Clostridium difficile* and their antimicrobial resistance to six antimicrobial classes, in a variety of fresh vegetables sold in retail in Ohio, USA, and (ii) conducted cumulative meta-analysis of reported prevalence in vegetables since the 1990s. Six antimicrobial classes were tested for their relevance as risk factors for *C. difficile* infections (CDIs) (clindamycin, moxifloxacin) or their clinical priority as exhaustive therapeutic options (metronidazole, vancomycin, linezolid, and tigecycline). By using an enrichment protocol we isolated *C. difficile* from three of 125 vegetable products (2.4%). All isolates were toxigenic, and originated from 4.6% of 65 vegetables cultivated above the ground (*n* = 3; outer leaves of iceberg lettuce, green pepper, and eggplant). Root vegetables yielded no *C. difficile*. The *C. difficile* isolates belonged to two PCR ribotypes, one with an unusual antimicrobial resistance for moxifloxacin and clindamycin (lettuce and pepper; 027-like, A^+^B^+^CDT^+^; *tcdC* 18 bp deletion); the other PCR ribotype (eggplant, A^+^B^+^ CDT^−^; classic *tcdC*) was susceptible to all antimicrobials. Results of the cumulative weighted meta-analysis (6 studies) indicate that the prevalence of *C. difficile* in vegetables is 2.1% and homogeneous (*P* < 0.001) since the first report in 1996 (2.4%). The present study is the first report of the isolation of *C. difficile* from retail vegetables in the USA. Of public health relevance, antimicrobial resistance to moxifloxacin/clindamycin (a bacterial-associated risk factor for severe CDIs) was identified on the surface of vegetables that are consumed raw.

## 1. Introduction

On September 16, 2013, the CDC declared in its Antimicrobial Resistance Threats Report spore-forming* Clostridium difficile* as a threat with “urgent” concern to public health in the USA [1] due to (i) the identification of antimicrobial resistance among human and food/animal derived* C. difficile* isolates to drugs commonly used in humans (particularly fluoroquinolones, e.g., moxifloxacin) and (ii) their increased rates of associated patient mortality since the early 2000s [1].* Clostridium difficile* was the only spore-forming organism considered as an antimicrobial threat, not because* C. difficile* infections (CDIs) are becoming difficult to treat with anti-CDI-treatments, but because* C. difficile* have increasing resistance to antimicrobials commonly used to treat other medical conditions in humans (e.g., moxifloxacin/clindamycin), which allows* C. difficile* to grow in the gut opportunistically causing resilient and severe CDIs.

Despite the recognition of frequent outbreaks of severe infections and new hypervirulent* C. difficile* strains since the mid-2000s [2], the major sources of infective spores remain poorly understood. Because the same hypervirulent strains were isolated from animals and retail meats in 2006 [3, 4], concerns about food contamination and foodborne transmission have emerged. Antimicrobial resistance to fluoroquinolones in food/animal isolates also emerged concurrently [5, 6].

Highlighting that the source of infection for most CDIs remains unknown, Eyre et al. conducted genome sequencing of isolates of human origin from hospitals in the UK in 2013 and discovered that patient-to-patient transmission accounts only for a small fraction of new nosocomial CDIs (<25%), compared to methicillin-resistant* Staphylococcus aureus* which was highly transmissible [7]. More recently, a complementary analysis of antimicrobial susceptibility and multilocus sequence typing of meat- and human-derived* C. difficile* isolates in Belgium showed that meat/human strains clustered within the same lineage, especially highly prevalent PCR ribotypes 078 and 014 [5, 8]. Together these findings highlight the need to improve our understanding of the ecology and potential transmission routes for this pathogen.

*Clostridium difficile* survive as spores in the environment on inert surfaces, animal feces, or contaminated farm soils for months or even years [9].* C. difficile* has also been isolated from retails vegetables since the 1990s [10–12], and their consumption has been proposed as a potential route for foodborne transmission [10, 12], but no information is available on its prevalence in vegetables in the USA. Similarly, the patterns of antimicrobial resistance for vegetable derived isolates are not well understood. The objectives of this study were to (1) investigate the prevalence of* C. difficile* in a sample of retail vegetables available in Ohio, (2) conduct minimum inhibitory concentrations (MIC) analysis of antimicrobial resistance for isolated strains compared to local referent isolates, and (3) determine the changes of pooled prevalence of reported* C. difficile* prevalence in vegetables since 1990s using cumulative meta-analysis.

## 2. Materials and Methods

Based on sample size estimations to achieve a study power of 0.8 targeting the 7-8% prevalence reported in vegetables in Scotland (STATA, v13.1) [12], we collected 125 vegetable product samples from 4 different retailers in Ohio, USA. Single units were systematically selected for purchase to include most classes of vegetables available in each retailer at the time of sampling (2 store chains, 1 auction market, and 1 in-farm market, <200 km apart). Under the assumption that the spores of toxigenic* C. difficile* may be present in agricultural soils, as previously reported in the United Kingdom [10] and Zimbabwe [13], we purposively selected product units having obvious soil residue on their surface. The products originated from the USA (CA, PA, OH, MI, WI, NY, and FL) and Mexico; however, for 20% of tested produce the origin was unknown.

Samples were transported intact inside sterile whirl pack bags in coolers with crushed ice until processing in the laboratory. Portions of 15 g of edible parts of the produce, including surface cuts (<3 mm thick) and/or the outermost leaves, were aseptically cultured for* C. difficile* by enrichment-based culture methods within 24 hours of collection. The enrichment (50 mL of selective broth with cycloserine and cefoxitin) and subsequent isolation steps were performed as described [6, 14]. In brief, samples were mixed with 50 mL of enrichment broth prepared with the ingredients for a commercial* C. difficile* agar (Oxoid) without the addition of agar and supplemented with 0.1% of sodium taurocholate and 0.05% L-cysteine. Following 7 days of anaerobic incubation at 37°C and centrifugation, the sediments were alcohol shocked with 99% ethanol in 1 : 1 v/v for 50 minutes at 23°C. Broth sediments were streaked onto* C. difficile* agar supplemented with cycloserine and cefoxitin. Because fluoroquinolones are one of the most commonly used antibiotics associated with a greater risk for CDI in humans, we did not use moxalactam and norfloxacin (a fluoroquinolone) supplementation as has been previously done in other culture studies because its use seems to introduce selection bias towards fluoroquinolone-resistant isolates as early noticed [5, 6, 14].

Following 5 days of anaerobic incubation, 365 nm UV-fluorescence was used to screen selective agar plates for suspect colonies. Biochemical confirmation was based on L-proline aminopeptidase activity (Pro Disc, Remel, Lenexa, KS, USA) and gene markers of toxin production (*tcdA*,* tcdB*,* cdtA*,* cdtB*, and* tcdC*) and* tpi*,* cdU*,* tcdE*, and* 16S* based on Lemee and Persson protocols [15, 16]. Strain PCR ribotyping was performed using the Bidet method [17]. Antigenic confirmation of toxigenicity was verified on the isolates using two ELISA kits, one for toxins A/B and another for toxin A (TechLab/Wampole), and Vero cell cultures using products and reagents exactly as previously described [18], ensuring the prevention of refrigeration-induced false-positive reactions as recently reported [19].

All antiseptic measures were in place to prevent sample cross-contamination in the laboratory as deemed necessary for* C. difficile* studies on food safety [20]. In addition, the fire-resistant countertop used during sample processing was in the following order: (i) swabbed every 5–10 samples with autoclaved unscented commercial cloths (Swiffer, 10 × 15 cm) premoistened with 5 mL of phosphate buffer saline, (ii) disinfected with 10% bleach, then with 70% ethanol, and (iii) finally flamed with a laboratory torch at a pace we had proved that eliminates spores of* C. difficile* PCR ribotypes 078 and 027 (common strains identified in foods and animals). All swiffers were stored at 4°C for 24 h and then enriched/cultured for* C. difficile* as described above for food samples. As a positive control we used* C. difficile* strain ATCC 9689. To prevent identification bias, all samples were recoded and concurrently tested in a blinded fashion.

Antimicrobial susceptibility testing was performed using commercial *E*-test strips (AB Biodisk, Solna, Sweden). The MICs against six antimicrobial classes of clinical relevance in humans were determined as described [18] for (i) metronidazole and vancomycin being first choice treatments for* C. difficile* infections (CDIs) in humans [21], (ii) moxifloxacin and clindamycin, widely associated with CDI induction [21, 22], and (iii) linezolid and tigecycline being recent therapeutic antimicrobial classes against CDI [23, 24]. In brief, 24-hour-old* C. difficile* colonies grown on blood agar resuspended in* Brucella* broth (Oxoid, Columbia, MD) were used to lawn prereduced* Brucella* agar plates to determine the susceptibility to the *E*-test strips after 48 hours of anaerobic incubation at 37°C, following the guidelines and breakpoints from the Clinical and Laboratory Standards Institute or reported MICs from previous studies [5, 24–26]. We chose to use moxifloxacin in this study as a fluoroquinolone representative, because it is one of the agents in this class (along with gatifloxacin) that has the lowest rates of bacterial resistance and well-established breakpoint criteria [14, 27].

To contextualize our prevalence findings we performed a meta-analysis of reported prevalence of* C. difficile* in vegetables in the past twenty years, following a random effects model and both pooled and cumulative statistics using the DerSimonian and Laird approach (STATA, v.13) [28]. Electronic search of literature was conducted in PubMed and Scopus bibliographic databases in May 2014 following established guidelines [29]. The search was repeated on November prior to publication of this paper. A search algorithm consisting of pathogen related terms (*Clostridium difficile*) and 32 vegetable terms was used. The detailed search algorithm, along with the inclusion, exclusion, and data extraction criteria are available as Supplementary Material at http://dx.doi.org/10.1155/2014/158601. Relevant studies were identified as any peer-reviewed publication reporting prevalence data for* C. difficile* in vegetables. Search verification included hand-searching of reference list of the latest published study [30]. The relevance screening was performed by two reviewers. Ad hoc grey literature search was conducted in Google search engine. Extracted data included prevalence, sample size, vegetable types, geographic region,* C. difficile* genotype, antimicrobial resistance, and culture methods. To allow for proper identification of accurate data and pooled estimations, only peer-reviewed published studies were included [31].

We used meta-analysis to synthesize published laboratory findings across regions to determine probability estimates of the overall prevalence and confidence intervals of* C. difficile* in vegetables. Cumulative meta-analysis is an approach that measures how the overall estimate changes over time as new published data become available [28]. Prevalence and 95% confidence interval (CI) estimates were computed for each study using the Wilson method because it produces CIs above zero when the study prevalence is near zero [32]. Because zero values prevent pooled estimations, for studies with zero prevalence, a smoothing value of 0.1 (1/5 of default value recommended [28]) was added to the number of events and total number of samples for minimal impact on prevalence and CIs while allowing pooled meta-analysis estimations (Wilson method with correction for continuity) [28, 33, 34]. The assumption for data normalcy was fulfilled by log-transforming the proportion estimates. Study heterogeneity was tested using *I* ^2^ statistics based on measure analysis for the deviations for each within-study variance from a central estimate for the collective between-study variance distribution [28]. Although publication bias is a common limitation of meta-analysis [35], estimations were not pursued due to the lack of a prepublication registry of prevalence based studies in food research to know how many studies start and do not get published.

## 3. Results

Following the enrichment protocol we isolated* C. difficile* from three of 125 products (2.4%; Table 1). All three isolates originated from above-ground vegetables (lettuce, green pepper, and eggplant, 4.6%). They all were ELISA positive for both toxins A/B and toxin A tests and were toxigenic to Vero cells. The isolation of* C. difficile* by enrichment indicates that the spore load on contaminated vegetables was at least 1 spore per 15 g of product. Quality control incubation of 17 countertop swabs was negative indicating the absence of cross-contamination during sample processing.

**Figure 1:**
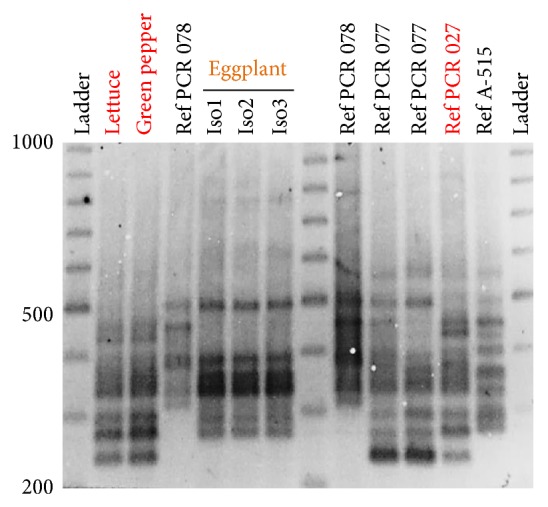
Toxigenic* C. difficile* strains isolated from fresh vegetables in Ohio. Illustration of PCR ribotyping of isolates and reference strains. This is the first isolation of PCR-027-like* C. difficile* from a sample of food or animal origin in the laboratory where this study took place. Laboratory cross-contamination was here quantified and thus deemed extremely unlikely. Reference strains PCR 027, 077, and 078 correspond to original isolates published by Rodriguez-Palacios et al. in 2006 which were characterized as relevant to the early 2000s epidemic outbreaks of CDIs in humans by Dr. J. Brazier at the Anaerobe Reference Laboratory for* Clostridium difficile* in Cardiff, UK [5]. All other reference strains correspond to isolates previously identified in animals in the USA [18, 37, 38].

**Figure 2:**
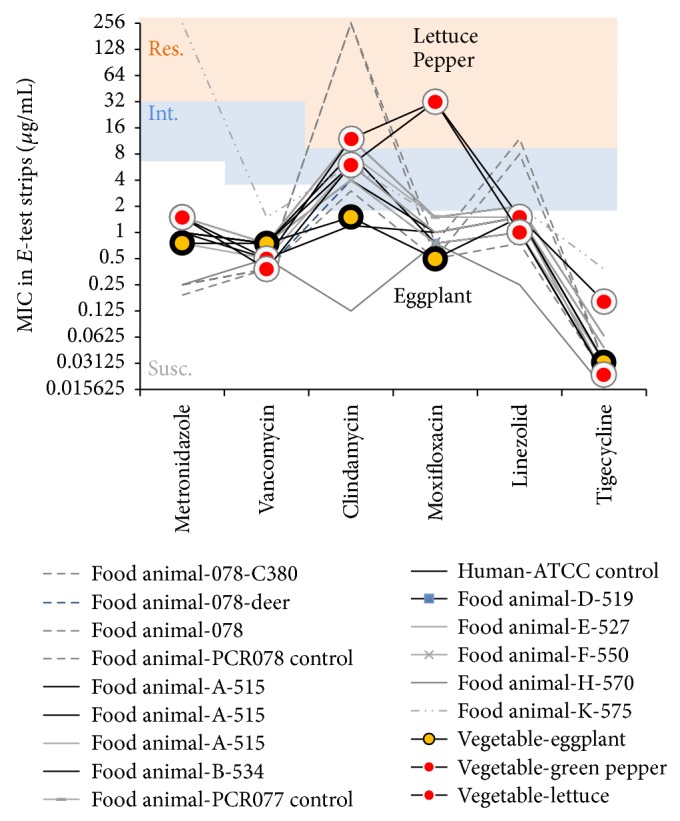
Visual representation of MIC values and distinct antimicrobial resistance patterns of three vegetable isolates (Figure 1) and 15 animal derived isolates previously characterized in the USA [18, 37, 38]. The thick black lines/circles connect MIC values for each of the three vegetable isolates. Thin lines represent a collection of historical isolates and quality control duplicate testing. Res., resistance; Int., intermediate; Susc., susceptible highlighted standard or published breakpoints.

PCR ribotyping showed that isolates belonged to two distinct PCR ribotypes. One isolate had no match to our collection of* C. difficile* isolates of animal origin, but two isolates belonged to the same PCR ribotype (indistinguishable from reference strain PCR ribotype 027 [5], Figure 1) and had an uncommon combination of antimicrobial resistance against moxifloxacin and clindamycin compared to our historic representative collection of food/animal derived isolates (Figure 2). These resistant strains were isolated from outer leaves of one iceberg lettuce sample and the surface of a green pepper (toxin profile: A^+^B^+^CDT^+^;* tcdC* 18 bp deletion). Despite their PCR ribotyping similarity, the two isolates had slightly different levels of susceptibility for tigecycline. The eggplant-derived isolate (A^+^B^+^ CDT^−^; classic* tcdC*) was susceptible to all antimicrobials.

A total of 34 references were retrieved via electronic search for the meta-analysis. After deduplication, 25 studies were screened for relevance, with two additional references identified through search verification process. After relevance screening five peer-reviewed studies were identified that reported prevalence of* C. difficile* in vegetables [10–12, 30, 39]. One unpublished study presented as a poster in 2012 reported zero prevalence of* C. difficile* among only 3 ready-to-eat salads and 5 sprout samples tested in Europe [40]. More recently, another unpublished study reported* C. difficile* in hospital food items (processed as mixed-meal homogenates) offered to patients admitted without gastrointestinal disease in a US hospital (0.2% for a pooled category reported as “vegetables, grains, and other” category) [41]. Another two studies on hospital meals in the USA have been reviewed [42] but remained unpublished. Because these studies did not fulfill the peer-reviewed publication criterion or provide study details, they were excluded from meta-analysis.

**Figure 3:**
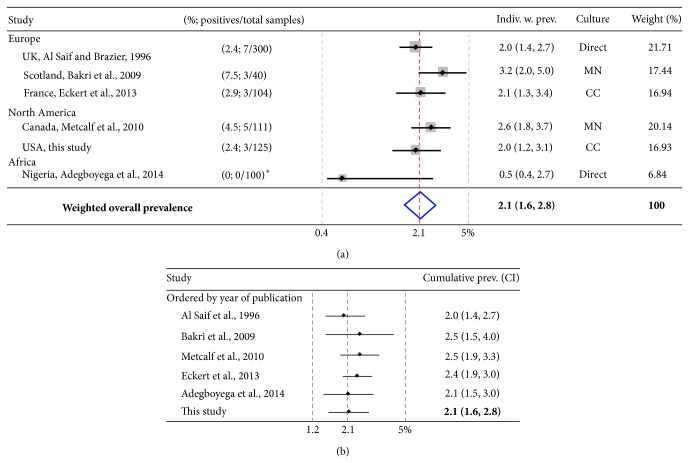
Systematic review and meta-analyses of* C. difficile* prevalence in vegetables for studies published between 1996 and 2014. (a) Forest plot of pooled meta-analysis with individual weighted estimates. Direct, MN, and CC indicate direct culture without enrichment, enrichment with moxalactam and norfloxacin (fluoroquinolone), or cycloserine/cefoxitin. With the inclusion of the Nigerian study [39], the (*I* ^2^) study heterogeneity increased from low 7.7% to moderate 62.2% using cut off criteria as described [28]. Prior to its inclusion, the weighted overall prevalence of* C. difficile* was still very similar 2.3% (1.9, 2.6) to what is here reported with 6 studies. Asterisk: see methods for how zero prevalence studies were handled to enable the estimation of pooled parameters; study u**s**ed direct contact culture plating on Brazier CCEY Agar as described in first report of* C. difficile* in vegetables [10]. (b) Cumulative meta-analysis depicting changing estimated prevalence trend as studies become available. Note the comparable homogeneity across studies and the similar overall weighted prevalence. The inclusion of the Nigerian study as described in methods [33] had little effect on final cumulative prevalence (2.3%, CI = 1.9, 2.8, before inclusion).

All identified studies were published after 1990. The origin and the date for each study are shown in Figure 3. The overall estimate of* C. difficile* prevalence in vegetables as estimated with meta-analysis was 2.1% (95% CI = 1.6, 2.8; Figure 3(a)). Cumulative meta-analysis showed that the* C. difficile* prevalence in vegetables has been continuously low over time (2.1%; Figure 3(b)). Although there was no significant heterogeneity across study prevalence values for 5/6 studies (except a Nigerian study [39]; chi-squared *P* = 0.36; *I* ^2^ heterogeneity of 7.7%; and between-study Tau-squared = 0.0034), metaregression was not conducted to quantify associations between variables due to the limited number of studies and sampling and culture variability.

## 4. Discussion

Because microbial food contamination may occur clustered at the production or processing site, the main goal of the present screening study was to determine if* C. difficile* could be isolated from a sample of a variety of retail vegetables in Ohio and not on an individual product type, which was the purpose of a previous study dedicated to salads in Scotland [12]. With our reported approach, which is similar to that of previous studies [10, 11], we have isolated toxigenic* C. difficile* from the outer leaves of one iceberg lettuce and the surfaces of an eggplant and a green pepper in Ohio. The prevalence of* C. difficile* in our sampled vegetables was 2.4%, which is similar to what has been recently reported in Canada, France, and Scotland and earlier in the UK (2.4–7.5%) [10–12, 30]. Our finding was also similar to our estimated pooled meta-analysis weighted prevalence of* C. difficile* in vegetables of 2.3% (2.0–3.2%; five studies). Cumulative meta-analysis indicated that the reported prevalence of* C. difficile* in vegetables has been homogeneous and similar over time (2.3%). Contrary to the expectations and knowledge that* C. difficile* spores survive in soils, none of the root vegetable products covered with soil debris in the present study were contaminated with* C. difficile*. Together, our prevalence findings should be interpreted cautiously because* C. difficile* spores can reach vegetables via various sources like contaminated manure, soils, or irrigation waters [43] as well as downstream of the vegetable production chain through worker hands and contamination in transit and retail, from storage displays or customers. In addition, most studies, including ours, used only one enrichment replicate. Increasing the number of culture method replicates may yield higher rates of* C difficile* in vegetables as it has been shown to increase the ability to detect* C. difficile* and the strain diversity in meats [14].

Irrespective of our PCR-genotyping findings, the significance of the presence of antimicrobial resistant* C. difficile* spores on vegetables remains uncertain because the food safety relevance with respect to spore load in foods and the link to CDI in susceptible humans is still not well understood. However, because CDI is often caused by* C. difficile* strains with high antimicrobial resistance to commonly used antimicrobials, it is relevant to highlight that 2/3 of our isolates in this study came from ready-to-eat vegetables (lettuce, pepper) and were resistant to moxifloxacin and clindamycin, both widely used as therapeutics in humans. Antimicrobial resistance of* C. difficile* is important because the use of antimicrobials increases the risk to CDI by disrupting the intestinal flora, augmenting the experimental susceptibility to colonization with low numbers of spores [44].

Antimicrobial MIC values, commonly used as decision-making tools, are often reported as a list of parameters (MIC_50_, MIC_90_, mean/SD, and % of resistant isolates) without interclass correlations for tested drugs. By conducting a breakpoint MIC-annotated line-plot analysis in this study we were able to visualize the distinct antimicrobial resistance for moxifloxacin and clindamycin in* C. difficile* isolates from vegetables compared to animal derived strains isolated by our laboratory from Ohio and other states in the USA [18] (Figure 2). Analysis of *E*-test MIC data from a previous study of* C. difficile* in ready-to-eat salads in Scotland (for metronidazole, vancomycin, clindamycin, and moxifloxacin) [12] matched our moxifloxacin/clindamycin findings, indicating that such clinically relevant resistance pattern may be frequent in vegetables.

Because most studies on vegetables have reported* C. difficile* strains relevant to human CDIs (same genotypes, toxin virulence, and antimicrobial resistance), preventive and educational measures are needed to reduce the risk of inadvertent exposure among susceptible populations as suggested [45]. Proper cleaning with removal of the outer leafy layers of fresh vegetables for raw consumption and adequate cooking and handling [46, 47] of relevant products might be beneficial to reduce foodborne exposure. In conclusion, the prevalence of* C. difficile* in vegetables remains low. Our report primarily emphasizes the presence of an antimicrobial moxifloxacin/clindamycin resistance combination in* C. difficile* isolated form ready-to-eat vegetables in the USA.

## Supplementary Material

The detailed search algorithm, along with the inclusion, exclusion, and data extraction criteria.

## Figures and Tables

**Table 1:** Retail fresh vegetables tested for *Clostridium difficile* in Ohio, USA.

Part of plant	Vegetable category	Risk^*^	*n*	*C. d* *if* *fi* *ci* *le* ^§^
Aboveground vegetables, *n* = 65	Leafy (lettuce/spinach/chard/herbs)	1	41	**1**
	Tomato/pepper/eggplant	2	13	**2**
	Berries	2	5	—
	Broccoli	—	3	—
	Green beans	—	3	—

Contact with soil, *n* = 13	Melons	2	3	—
	Cucurbits	3	10	—

Root vegetable, *n* = 39	Onions	2-3	20	—
	Carrots/potatoes/beets/parsnip	3	19	—

Other, *n* = 8	Sprouts	2	2	—
	Mushrooms	—	6	—

Total			125	3 (2.4%)

^*^Global priority levels for fresh produce safety assigned by the FAO/WHO [36]; priorities 2 and 3 vary across regions.

^§^Toxigenic isolates from conventional lettuce, eggplant, and green pepper.
