# Supplementary material for: Clostridium difficile with Moxifloxacin/Clindamycin Resistance in Vegetables in Ohio, USA, and Prevalence Meta-Analysis
Source: J Pathog. 2014 Dec 14;2014:158601. doi: 10.1155/2014/158601 (PMC4279118; doi:10.1155/2014/158601)
Supplement: Supplementary file 1 — The detailed search algorithm, along with the inclusion, exclusion, and data extraction criteria. [file 158601.f1.pdf]

SUPPLEMENTARY MATERIAL

Clostridium difficile with moxifloxacin-clindamycin resistance in vegetables in Ohio, USA, and a cumulative prevalence meta-analysis

Rodriguez-Palacios, Alex[1*]; Ilic, Sanja[2*] and LeJeune, Jeffrey T[3]

Supplementary table - Search terms, relevance screening, and data extraction strategy

**Search**		
**Criterion**	**Inclusion**	**Definition**
**Peer reviewed literature**	**Clostridium difficile**	**Search terms:** C. difficile, clostridium, difficile
	**Vegetables**	**Search terms: vegetable(s) l**ettuce, spinach, cabbage, fresh leafy green herbs, endive, arugula, chard, watercress, radicchio, frizee, mustard green, beans, cauliflower, broccoli, celery, onion, cantaloupe, watermelon, melon, mushroom, carrot, potato, garlic, radish, corn, peas, cucumber, tomato, peppers, alfalfa, sprouts
	**Database**	**Search algorithm**
	**Pubmed**<br>**21 records retrieved**	(clostridium difficile) AND (vegetable OR lettuce OR spinach OR cabbage OR herbs OR endive OR arugula OR chard OR watercress OR radicchio OR frizee OR mustard green OR beans OR cauliflower OR broccoli OR celery OR onion OR cantaloupe OR watermelon OR melon OR mushroom OR carrot OR potato OR garlic OR radish OR corn OR peas OR cucumber OR tomato OR peppers OR alfalfa OR sprouts)
	**Scopus**<br>**12 records**	(TITLE-ABS-KEY(clostridium difficile) AND TITLE-ABS - KEY(vegetable OR lettuce OR spinach OR cabbage OR herbs OR endive OR arugula OR chard OR watercress OR radicchio OR frizee OR mustard green OR beans OR cauliflower OR broc coli OR celery OR onion OR cantaloupe OR watermelon OR me lon OR mushroom ORcarrot) OR TITLE-ABS- KEY(potato OR garlic OR radish OR corn OR peas OR cucumb er OR tomato OR peppers OR alfalfa OR sprouts))
**Criterion**	**Inclusion**	**Definitions**
**Relevance screening of titles and abstracts (RS1)**		
Study topic	Studies investigating:<br>**Prevalence** of<br>***Clostridium difficile*** in<br>**Fresh or frozen vegetables**	Occurrence of two or more cases of a similar illness as a result of persons ingesting the same food.<br>One or more cases of disease that occur in a scattered or unpredictable manner. (<br><br>Vegetables (either conventional or organic), including those that may have been peeled or cut but otherwise remain in their original physical form and are kept in a fresh (minimally processed) or frozen state intended for raw consumption.
Time period	**Not limited**	Studies published in any time period
Geographic location	**Not limited**	**Studies originating from any geographic location**
Study type	**Primary research**	**Peer**-reviewed publications or publicly available reports describing data collected and analyzed by the authors.
Study language	Not limited	Studies published in any language

Supplementary table - Search terms, relevance screening, and data extraction strategy
(cont…)

**Relevance screening of full articles (RS2)**		
Confirmation of relevance	Same categories as described under RS1 when the relevance was not clear based on abstract only	Same definitions as above
**Data Extraction**		
Data	Prevalence data	raw or unadjusted prevalence data with both the numerator and denominator reported or proportion and either numerator or denominator;<br>or, unadjusted effect estimates with reported sample size and measure of variability, or adjusted effect estimates, sample size, measure of variability, and exact *P*-values were considered essential.
Date	**Day/Month/Year**	Study date
Study origin	**Country, State/Province, City/Town**	
Clostridium difficile	**Classification**<br>**Culture protocol**	Ribotype, Toxin production, other
Produce	**Produce category**<br>**Produce name**<br><br><br><br><br><br><br><br>**Produce state**<br>**Special type**<br>**Growing conditions**<br><br>**Country of origin**	**Produce grown close to the ground:** leafy greens: spinach, lettuce, arugula, escarole, endive, cabbage, kale, fresh leafy herbs (basil, cilantro, parley, mint), alfalfa sprouts, bean sprouts, cauliflower, broccoli, celery, green onions, cantaloupe, watermelon, melon, mushrooms.<br>**Produce grown in the ground:** root vegetables: carrot, onion, potato, garlic, radish<br>**Not peeled vegetables**: cucumber, tomato, green beans, other beans, peppers.<br>**General terms:** produce, vegetables, mixed mixed vegetables, leafy salad, other produce and “not-specified”.<br>**Fresh or frozen** produce.<br>**Organic or conventional** produce.<br>Produce grown in the **field** or in the **greenhouse**.<br>Reported country of origin of implicated produce. **Country, State/Province, City/Town**
